# Divergent microtubule-organizing center strategies in mammalian gametogenesis

**DOI:** 10.1016/j.treopn.2025.12.002

**Published:** 2026-02-18

**Authors:** Marnie W. Skinner, Philip W. Jordan

**Affiliations:** 1 Department of Biochemistry and Molecular Biology, Johns Hopkins University Bloomberg School of Public Health, Baltimore, MD, USA; 2 Department of Biochemistry and Molecular Biology, Uniformed Services University of the Health Sciences, Bethesda, MD, USA; 3 The Henry M. Jackson Foundation for the Advancement of Military Medicine, Bethesda, MD, USA; 4 School of Biomedicine, The University of Adelaide, Adelaide, South Australia, Australia

## Abstract

Meiotic chromosome segregation relies on the spindle microtubules, which are assembled by microtubule-organizing centers (MTOCs). Mammalian gametogenesis reveals starkly divergent strategies between the sexes. Spermatocytes harbor canonical centrosomes that undergo biogenesis events unique to the meiotic cycle. However, recent findings reviewed here show that these centrosomes are dispensable for chromosome segregation and that a noncentrosomal MTOC pathway can accomplish this segregation. By contrast, oocytes eliminate centrioles and assemble microtubule spindles by coalescing pericentriolar material components to form bipolar MTOCs, a process assisted by a chromosome-driven spindle assembly pathway. This review discusses the species-specific differences in MTOC regulation between mouse and human oocytes and examines how defects in these MTOC pathways are a primary cause of aneuploidy and infertility.

## Setting the stage for healthy gametes

Sexual reproduction hinges on the successful formation of healthy, haploid gametes—spermatozoa and oocytes. Haploid gametes are produced when a diploid cell undergoes **meiosis** (see Glossary), a highly specialized cell division program that involves a single round of DNA replication followed by two consecutive rounds of chromosome segregation. The fidelity of meiosis is paramount; errors in chromosome segregation lead to gamete aneuploidy, which is a primary cause of embryonic failure, congenital disorders such as Down syndrome, and infertility [1]. The clinical burden is substantial, with an estimated 17.5% of the global adult population experiencing infertility, a condition in which failures in gametogenesis are a significant contributing factor [2].

The successful execution of meiosis depends on a series of precisely orchestrated molecular events. In the first meiotic division (meiosis I), homologous chromosomes are segregated, while the second division (meiosis II) separates sister chromatids, akin to a mitotic division [3]. To achieve euploid gamete formation, the cell must first establish robust physical linkages between homologous chromosomes through genetic recombination and then assemble a bipolar microtubule (MT) spindle [4]. The dynamic MT spindle apparatus, composed of tubulin polymers, generates the precise forces required to align and then segregate the chromosomes accurately to opposite poles of the cell [5,6]. The spatial and temporal controls of spindle assembly are governed by **microtubule-organizing centers (MTOCs)**, which serve as the primary sites for MT nucleation [7].

The establishment of physical linkages between homologous chromosomes, known as chiasmata, is a complex process that occurs during meiotic prophase I. Homologous chromosome interactions are initiated by the programmed creation of 200–300 DNA double-strand breaks by the SPO11–TOP6BL topoisomerase complex [8–10]. A complex repair machinery then uses the homologous chromosome as a template, ultimately resolving a subset of these breaks as crossover events [4,11]. To stabilize the chromosome pairs during homologous chromosome recombination, a proteinaceous structure called the synaptonemal complex (SC) assembles between them [12,13]. Once the double-strand breaks are repaired, the SC disassembles, and the homologous chromosomes are held together via crossover recombinations, which are known as chiasmata. At this point, the chromosomes are ready to engage with the meiotic spindle. MT spindle interactions with chromosomes are mediated by kinetochores, large protein complexes assembled at the centromeres that serve as the direct attachment points for spindle MTs [14]. The cell employs a rigorous surveillance system, the spindle assembly checkpoint, which ensures that division does not proceed until every chromosome is properly attached and under tension from opposing spindle poles [15]. The chiasmata are essential for generating this tension, thus demonstrating a critical dependency between the early events of recombination and the later fidelity of chromosome segregation [16].

The mechanisms driving MTOC function during mammalian gametogenesis are profoundly sexually dimorphic. Male germ cells employ the canonical, centriole-containing centrosome, which undergoes a complex and uniquely adapted duplication cycle to organize the spindles for both meiotic divisions [17,18]. The centrosome is a highly structured, template-driven process that ensures consistency and provides the foundation for the subsequent morphological changes necessary for a haploid round spermatid to transform into an elongated sperm harboring a functional flagella [19–21]. As such, defects in the MTOC during **spermatogenesis** often lead to spermatids with severe structural abnormalities [22].

In stark contrast to spermatocytes, mammalian oocytes undergo a programmed elimination of their centrioles early in development [23]. They must, therefore, devise an alternative strategy, assembling massive, functional meiotic spindles from a multitude of small, **acentriolar MTOCs (aMTOCs)**, which self-organize within the cytoplasm [24,25]. The challenge of acentriolar spindle assembly in females creates a different set of vulnerabilities, primarily related to spindle instability and the fidelity of chromosome segregation [26]. This is a major contributor to the high incidence of aneuploidy in human oocytes, which increases dramatically with maternal age [1,27].

This review aimed to explore the molecular mechanisms governing these divergent pathways of MT organization in both spermatogenesis and **oogenesis**. The review examines the architecture and biogenesis of both centrosomal and acentriolar MTOCs, the key protein players that regulate their function, and the evolutionary pressures that may have driven this divergence. The fundamental difference in MTOC strategy between the sexes is not merely a structural curiosity but a core determinant of their respective vulnerabilities. The centriole-driven process in males is susceptible to defects in the duplication and remodeling of its core components. However, these defects do not cause chromosome missegregation, and it was recently shown that mammalian spermatocytes rely on **noncentrosomal MTOCs (ncMTOCs)** to facilitate chromosome segregation during meiosis I and II [19,20]. Instead, centriole perturbations often manifest as structural pathologies, such as morphological defects during **spermiogenesis** and sperm motility issues. Conversely, the more fluid, self-organizing system in oocytes is inherently prone to errors in process—specifically, in spindle geometry and stability—contributing to the alarmingly high rates of aneuploidy observed in human eggs [24]. By connecting the fundamental biology of the meiotic spindle to its clinical consequences, a more nuanced understanding of reproductive failure can be achieved and new avenues for future investigation identified.

## Dual mechanisms of spindle assembly during male meiosis

The fidelity of spermatogenesis hinges on the precise segregation of chromosomes during two consecutive meiotic divisions. This crucial task is executed by the meiotic spindle, a bipolar array of MTs meticulously organized by MTOCs. For decades, the canonical centrosome has been considered the primary MTOC responsible for spindle assembly during mammalian spermatogenesis. However, emerging research reveals a more complex system in spermatocytes, where an ncMTOC operates alongside, and can even compensate for, the centrosome.

### Centrosome architecture and mitotic biogenesis

**Figure 1.**
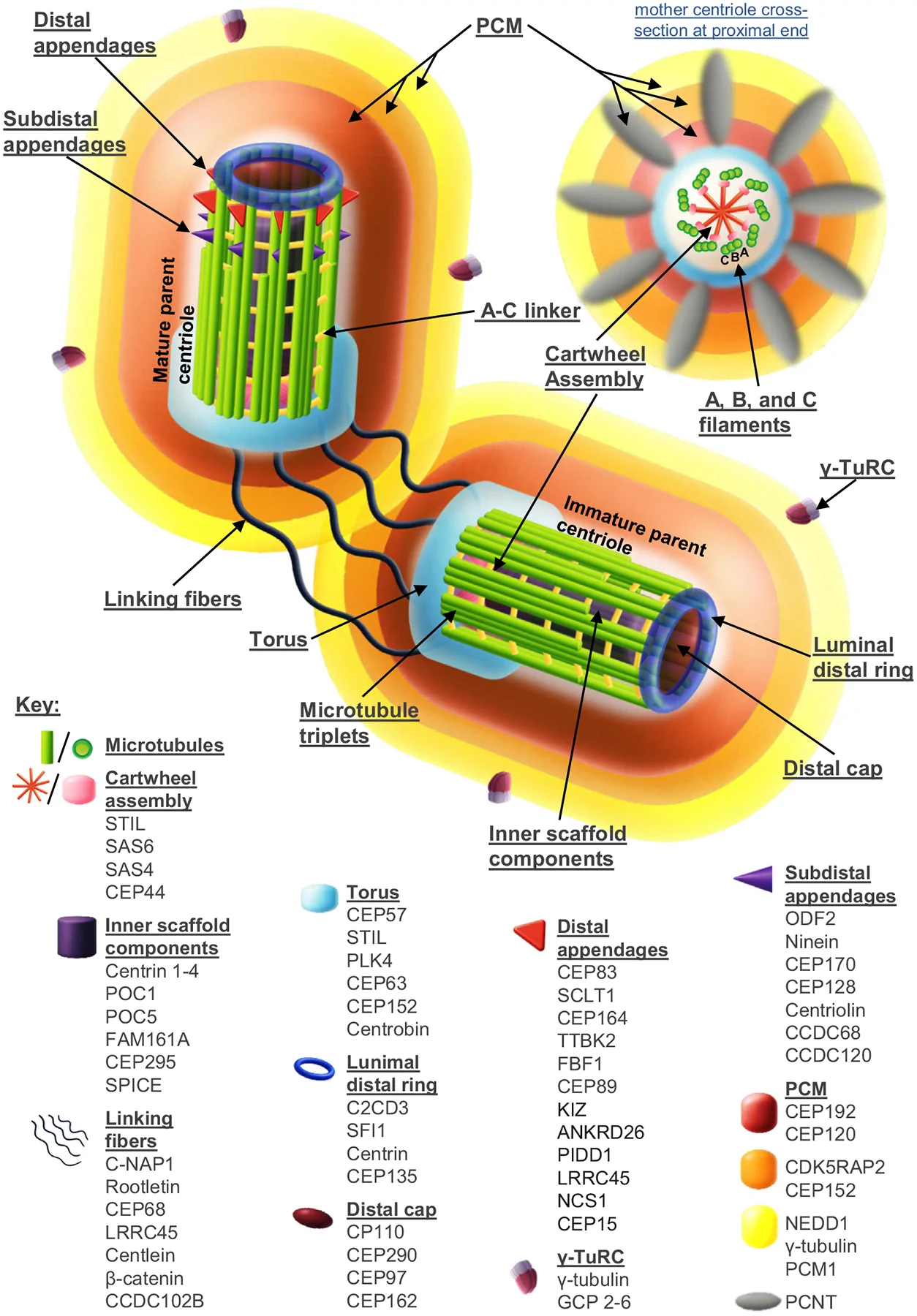
The centrosome structure. The centrosome consists of two centrioles and the surrounding proteins known as the PCM. The centrioles are composed of nine microtubule triplicates arranged in a cylindrical structure around a central cartwheel base at the proximal end. Inner scaffold components assemble in the middle and distal regions within the cylinder, and the luminal distal ring and distal cap proteins bind at the distal end of the centriole. The mature centriole harbors distal and subdistal appendages that facilitate PCM factor recruitment to the centrosome and membrane docking during ciliogenesis. The PCM assembles in an organized manner around the centrioles, with some proteins localizing closer to the centrioles, while others remain farther away, depending on their function. For additional information, refer to Box 1 and Tables S1 and S2. Image created with insight from a previous work [28]. γ-TuRC: γ-tubulin ring complex; PCM: pericentriolar matrix.

The canonical centrosome, the primary MTOC in many animal cells, consists of two cylindrical centrioles embedded in **pericentriolar material (PCM)** (Figure 1, Box 1, and Tables S1 and S2 in the Supplemental information online). Centrioles typically exhibit a ninefold symmetry of MT triplets that form a cartwheel-like structure [29]. These triplets are interconnected by linkers and anchored to a central proximal cartwheel structure, while the distal end is capped by proteins that must be removed for ciliogenesis [30,31]. Centriole maturation requires the acquisition of distal appendages, which are essential for forming cilia, and subdistal appendages, which are crucial for MT anchoring and recruiting PCM proteins [37,38]. The PCM is a highly ordered protein matrix that surrounds the centrioles, stabilizes them, and coordinates MT nucleation [39]. The two centrioles within a centrosome are tethered by linker fibers [53,54].

Centriole duplication is orchestrated by the master regulator kinase, **polo-like kinase (PLK)4** [60–62]. In mammals, PLK4 is activated by homodimerization and transautophosphorylation, enabling it to initiate duplication [60,62]. Immediately after centriole duplication, PLK4 is rapidly targeted for proteasomal degradation [60,62]. This precise temporal control, linking activation directly to subsequent degradation, ensures that only one duplication event occurs per cell cycle, thereby preventing centriole overduplication, which is often linked to genomic instability and tumorigenesis [60,62, 63]. Following duplication, the centrosome must undergo maturation and separation during the G2 phase [39]. The linker fibers are disassembled during the G2-to-mitosis transition by cell cycle kinases such as **PLK1** and **Aurora kinase A (AURKA)** to allow the kinesin-like protein **KIF11** to drive centrosome separation [56,64], a process critical for establishing the mitotic spindle, cell polarity, and successful cell division [58,59]. The roles of PLK1 and AURKA in mediating centrosome separation are conserved during spermatogenesis, as conditional mutations in *Plk1* and *Aurka* in mouse spermatocytes result in persistent monopolar spindles during meiosis I [17,18].

### A unique centrosome cycle in spermatogenesis

**Figure 2.**
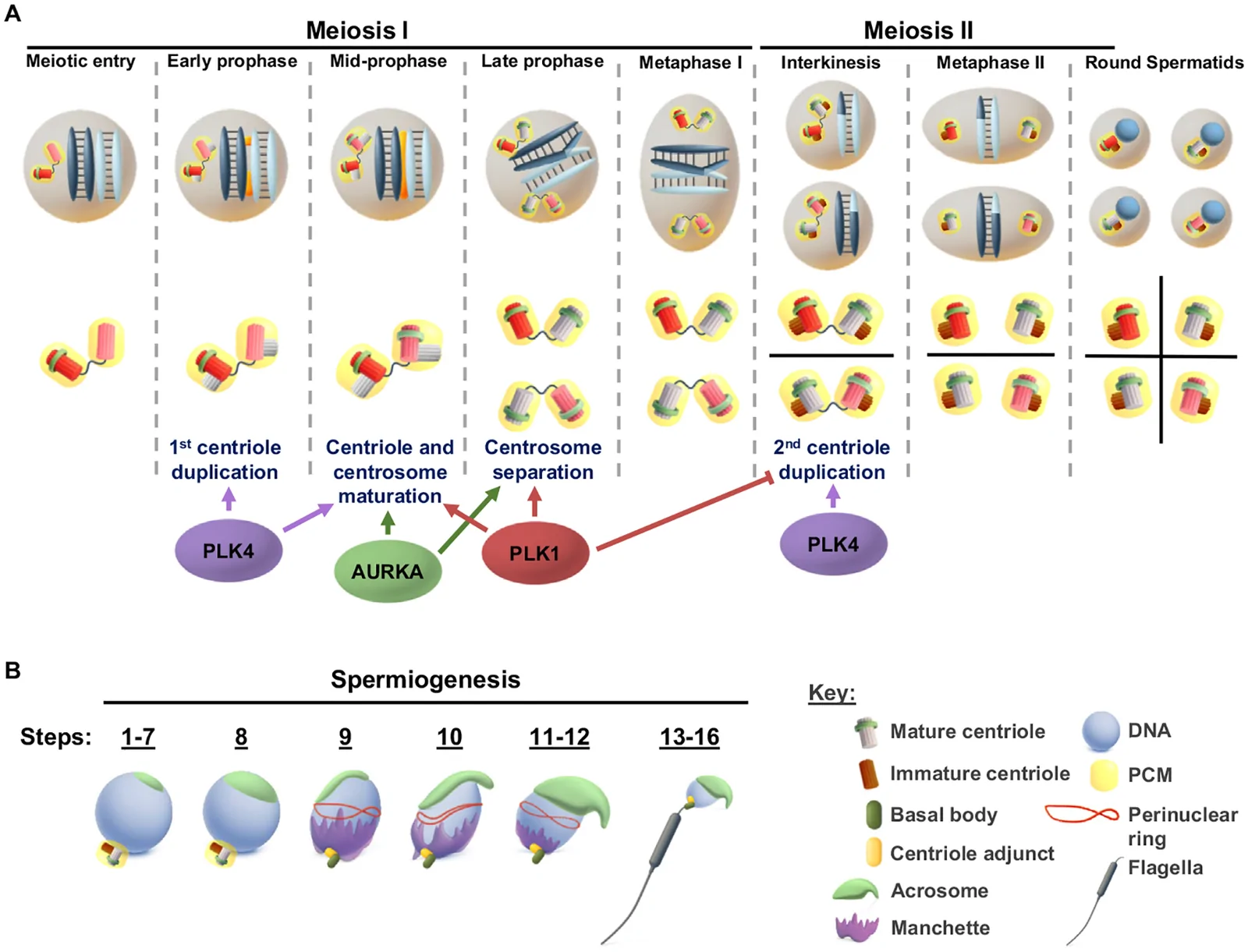
Centrosome biogenesis during spermatogenesis and spermiogenesis. (A) Centrosome biogenesis during spermatogenesis differs from mitotic centrosome biogenesis in timing and number. The first centriole duplication is uncoupled from S-phase and occurs during early meiotic prophase (leptotene stage). This is followed by the maturation of all three immature centrioles between mid-prophase and metaphase I. Centrosomes separate during diakinesis and migrate to form bipolar spindle poles at metaphase I. Following meiosis I, a second centriole duplication occurs during interkinesis. Each metaphase II spindle pole harbors a complete centrosome, ensuring that each round spermatid inherits one mature and one immature centriole. (B) Spermiogenesis is the postmeiotic transition of round spermatids to spermatozoa, consisting of four main phases: Golgi (steps 1–3), cap (steps 4–7), elongation (steps 8–12), and maturation (steps 13–16). In the Golgi phase, acrosomal proteins accumulate proximally, and centrioles attach distally to the nucleus. During the cap phase, the acrosome expands to cover ~50% of the spermatid’s surface. In the elongation phase, transient structures—the manchette and perinuclear ring—form to facilitate protein trafficking and designate the spermatid hemisphere. This elongates the head as the flagellum forms. In the maturation phase, these structures disassemble, and the DNA condenses. For additional information, refer to Box 2. AURKA: Aurora kinase A; PCM: pericentriolar matrix; PLK: polo-like kinase.

During spermatogenesis, spermatocytes undergo two consecutive meiotic divisions to produce haploid spermatids. Consequently, spermatocytes must also duplicate their centrioles twice to ensure a complete centrosome is present at each spindle pole during both metaphase I and metaphase II. While centriole duplication is tightly coupled with the initiation of DNA synthesis in mitotically dividing cells, this is not the case for spermatocytes. They enter meiosis with a single centrosome containing only two centrioles. Instead, the first round of centriole duplication is uncoupled from DNA replication and occurs during early prophase I, specifically during the leptotene stage (Figure 2A) [17–19]. This duplication event is dependent on PLK4. Inhibition or conditional knockout of PLK4 prevents this first centriole duplication event, highlighting its essential role [19]. Other proteins, such as the centriole component SAS4 and the WD repeat domain protein WDR62, are also required for this process, as they ensure the proper recruitment of essential centriole and PCM factors [20,65].

Following centriole duplication, the centrosome contains four centrioles, which then undergo a maturation process during mid-to-late prophase (pachynema to diakinesis) [17,19,20]. This maturation is critical for the centrosome to function as an MTOC. Maturation involves the sequential acquisition of distal appendages, marked by the protein CEP164. Initially, in pachynema, only the oldest parent centriole is mature. Then, the original immature parent centriole undergoes maturation in pachynema. By diakinesis, all four centrioles acquire distal appendages—a unique feature of meiosis, ensuring that each resulting spermatid can later form a flagellum [17,19,20]. This maturation process is heavily regulated by the kinases PLK1, PLK4, and AURKA. PLK4 is necessary to stabilize the immature parent centriole during meiotic prophase [19]. PLK1 is essential for the acquisition of distal appendages on the newly formed centrioles, while both PLK1 and AURKA are required for the recruitment of other key PCM components and the overall maturation of the centrosome [17,18].

During the transition from diplonema to diakinesis, two centrosomes, each containing a mature centriole pair, separate and migrate to opposite sides of the nuclear periphery [17–19]. This separation is necessary to facilitate bipolar spindle formation prior to chromosome segregation during meiosis I. This process also requires the coordinated action of PLK1 and AURKA, which facilitate the removal of the proteinaceous linker that holds the duplicated centrosomes together [17,18]. Mutations in centrosome components and overduplication of centrioles during meiosis can also lead to aberrant distribution of centrosome components and defects in spindle assembly, underscoring the importance of coordination of a network of proteins in this process [19,66].

Following the completion of meiosis I, during the brief stage of interkinesis, the second centriole duplication event occurs [17,18]. This event is again uncoupled from DNA synthesis. However, the newly formed centrioles do not recruit distal appendages and, instead, remain classified as immature. Immediately after centriole duplication during interkinesis, two centrosomes separate to form bipolar spindle poles in metaphase II, each harboring one mature and one immature centriole [17,19,20].

The resulting round spermatids produced after completing meiosis inherit one complete centrosome with one mature and one immature centriole [17,19,20]. This pattern of centriole inheritance is critical for the complex morphological changes that spermatids undergo to develop into functional spermatozoa (Figure 2B and Box 2). These postmeiotic remodeling processes, collectively known as spermiogenesis, rely on the two inherited centrioles to form the sperm flagellum and the head-to-tail coupling apparatus. Centriole remodeling differs between mammalian species (Box 2) [21,73,79,81,85]. Nevertheless, the failure to inherit a complete set of two centrioles results in severe defects in spermatid morphology and infertility.

While the understanding of the meiotic centrosome cycle has advanced, several aspects of its regulation remain intriguing and distinct from mitosis. Unlike in somatic cells, where PLK4 levels are tightly controlled through degradation, PLK4 remains present at the centrosome throughout spermatogenesis, suggesting that a unique, meiosis-specific mechanism of functional inhibition is at play [19]. PLK4 is the limiting factor for centriole duplication, as confirmed by experiments showing that its overexpression during meiotic prophase bypasses these inhibitory controls and leads to massive centriole amplification [19]. Furthermore, the temporal separation of the two duplication events is strictly enforced, with sufficient levels of PLK1 being crucial during prophase I to prevent premature licensing of the second duplication event that is meant to occur during interkinesis [17]. Perhaps, most surprisingly, centriole duplication failure does not cause aberrant chromosome segregation events as it often does in mitosis. Instead, spermatocytes demonstrate remarkable plasticity, forming proficient bipolar spindles and accurately segregating chromosomes by using an ncMTOC mechanism [19,20]. This reveals that while centrioles are indispensable for the ultimate formation of a functional spermatozoon (Figure 2B and Box 2), they are not strictly required for the meiotic divisions themselves. The precise molecular composition of this ncMTOC and the exact nature of the inhibitory factors that regulate PLK4 and PLK1 function during spermatogenesis remain key unanswered questions for ongoing research.

### Emerging complexity: the role of ncMTOCs in spermatocytes

**Figure 3.**
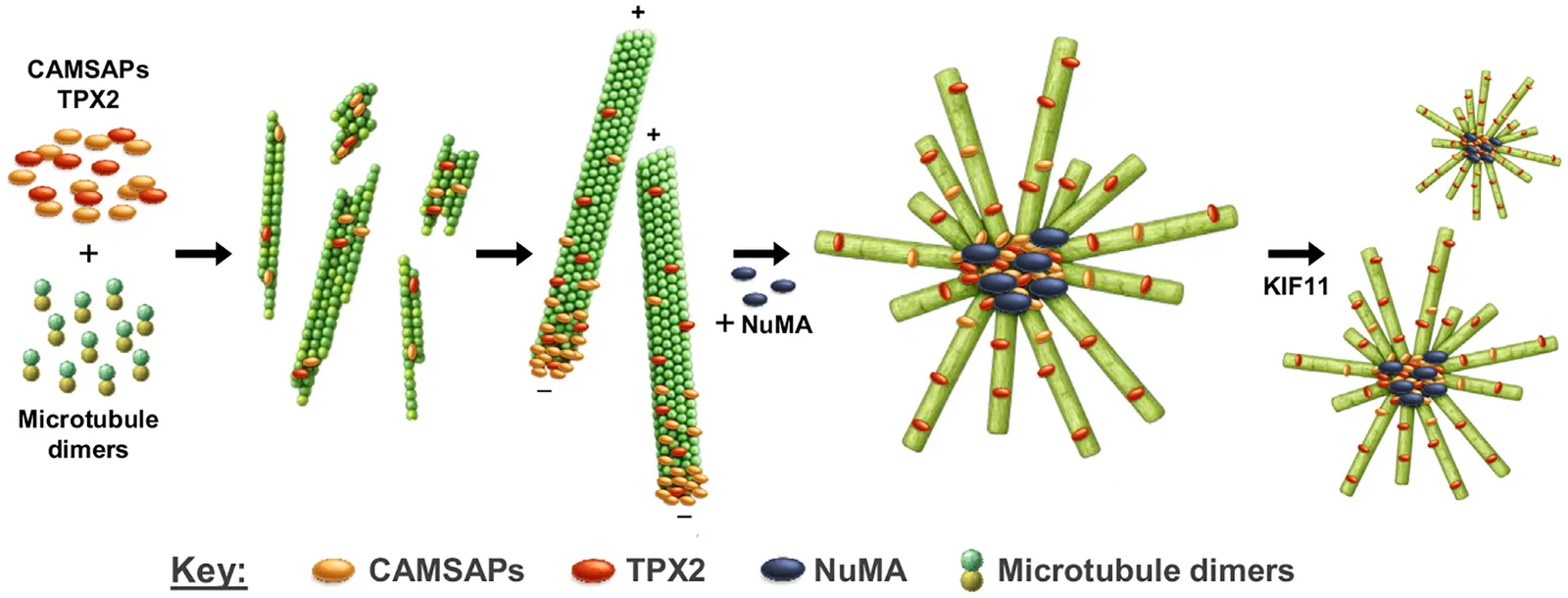
ncMTOC proteins coordinate microtubule assembly and bipolar spindle pole formation independent of the centrosome. Mammalian spermatocytes can complete meiotic chromosome segregation by using the ncMTOC proteins TPX2, NuMA, CAMSAP1–3, and KIF11 [19,20]. CAMSAP1–3 and TPX2 facilitate microtubule nucleation and stabilization. Microtubule organization and focusing are coordinated by NuMA. Finally, spindle pole separation into a bipolar orientation is facilitated by the molecular motor KIF11. CAMPSAP: calmodulin-regulated spectrin-associated protein; ncMTOC: noncentrosomal microtubule-organizing center; NuMA: nuclear mitotic apparatus protein; TPX2: targeting protein for Xklp2.

While the highly regulated centrosome cycle is central to male meiosis, recent evidence suggests a fascinating layer of additional complexity. Studies have identified the presence and activity of ncMTOCs in male meiotic cells, particularly in contexts where centrioles may be absent or functionally distinct [19,20]. ncMTOCs lack all centrosomal proteins, including the canonical MT nucleation protein γ-tubulin and associated **γ-tubulin ring complexes (γ-TuRCs**; MT nucleators containing GCP2–6) [25,90–92]. In the ncMTOCs, additional proteins have been implicated in promoting MT nucleation (Figure 3). These proteins include **calmodulin-regulated spectrin-associated protein (CAMSAP)1–3**, **targeting protein for Xklp2 (TPX2)**, and colonic and hepatic tumor overexpressed gene protein (ch-TOG) (Table S2) [83,93–97]. The CAMSAP proteins are classified as MT minus-end stabilizers that help prevent the disassembly of the MT minus end and have been observed to promote MT nucleation in the absence of γ-tubulin in microglial cells [83,98–100]. TPX2 is also an MT-stabilizing protein that binds along the lengths of MTs and promotes MT nucleation [100–102]. While not sufficient independently, the ch-TOG protein has been observed *in vivo* to promote γ-tubulin-independent MT nucleation synergistically with TPX2 [93]. Additional proteins implicated in mitotic ncMTOCs that aid in MT organization include **nuclear mitotic apparatus protein (NuMA)** and ribonucleic acid export (RAE)1. RAE1 facilitates NuMA–MT interactions as it binds with the N-terminal end of NuMA and MT structures. NuMA can also bind to MTs directly and acts to focus MT minus ends into unified locations [103–106]. It is most common to observe ncMTOCs in terminally differentiated cell types [90]. However, ncMTOCs have also been observed in male spermatocytes undergoing division without centrioles [19,20]. The ncMTOCs in spermatocytes harbor the proteins CAMSAP1–3, TPX2, NuMA, and KIF11, which all play roles that contribute to MT nucleation, stabilization, organization, and bipolar spindle orientation necessary during cellular division [19,20].

The coexistence of a canonical centrosomal pathway and an ncMTOC pathway in spermatocytes raises the question of whether both are required to ensure meiotic fidelity. Spermatogenesis is a long and complex process where a single point of failure in the primary MTOC system could be catastrophic for fertility. Based on recent work using conditional knockout mice, it was shown that ncMTOCs can mediate proficient chromosome segregation during spermatogenesis in the absence of centrosomes [19,20]. However, it is not yet clear whether centrosomes can facilitate chromosome segregation in the absence of ncMTOC components during spermatogenesis. On one hand, null mutations in *Tpx2, Numa1*, and *Kif11* result in embryonic lethality, necessitating conditional models to study their roles during gametogenesis [107–109]. On the other hand, it is known that mutations in *Camsap1, Camsap2*, and *Camsap3* in mice lead to fertility defects [83,96,110]. However, only mutations in *Camsap1* were shown to cause aberrancies during spermiogenesis [83], whereas this does not appear to be the case when *Camsap2* is mutated [83,110], and spermatogenesis has yet to be assessed for *Camsap3* knockout mice [96]. Because CAMSAPs are important for other ciliated cells, complete null mutations lead to health defects that can make the assessment of spermatogenesis difficult. Therefore, it will be important to assess their functions using conditional knockout of *Camsap1, Camsap2*, and *Camsap3* alleles to specifically deplete them during spermatogenesis. Furthermore, due to the similar localization pattern of CAMSAP proteins [19,83], there is a possibility of functional redundancy between these proteins. As such, there may be a need to create mice with double and triple conditional knockout *Camsap* alleles. The precise interplay between these two systems remains a critical area for future investigation and challenges the long-held view of the centrosome as the sole organizer of the male meiotic spindle.

## The female strategy: building a spindle from scratch

Meiotic spindle assembly in oocytes represents a radical departure from the centrosomal pathway of spermatocytes. By intentionally discarding its centrioles, the oocyte forgoes the use of a pre-existing template and, instead, embraces a remarkable process of self-organization, building a massive, functional bipolar spindle from a diffuse population of MTOC components. This acentriolar pathway, while essential for proper embryonic development, introduces unique vulnerabilities that contribute to the high rate of chromosome segregation errors in female meiosis.

### The purposeful elimination of centrioles

During mid-prophase of oogenesis, the developing oocyte systematically eliminates its centrioles [111–113]. This is not a sign of decay but a crucial evolutionary adaptation with at least two key purposes. First, it prevents parthenogenesis (development without fertilization), as the oocyte is rendered incapable of forming the MT asters needed for early embryonic cleavage on its own [113–116]. Second, it ensures that the resulting human zygote inherits only a single centrosome, which is delivered by the sperm upon fertilization [21,117,118]. This strict paternal inheritance of the primary embryonic MTOC is critical for maintaining genomic stability. The presence of more than one centrosome in the zygote would lead to the formation of multipolar spindles during the first mitotic divisions, causing catastrophic chromosome missegregation and embryonic death [119]. The elimination of the maternal centrioles is therefore a fundamental strategy to safeguard the genome of the next generation. In fact, genetic variations in human *PLK4* have been linked to causing centriole amplification or centriole defects in zygotes, which can lead to multipolar or monopolar spindles, chromosome missegregation, and embryonic lethality [120,121].

### The rise of aMTOCs

**Figure 4.**
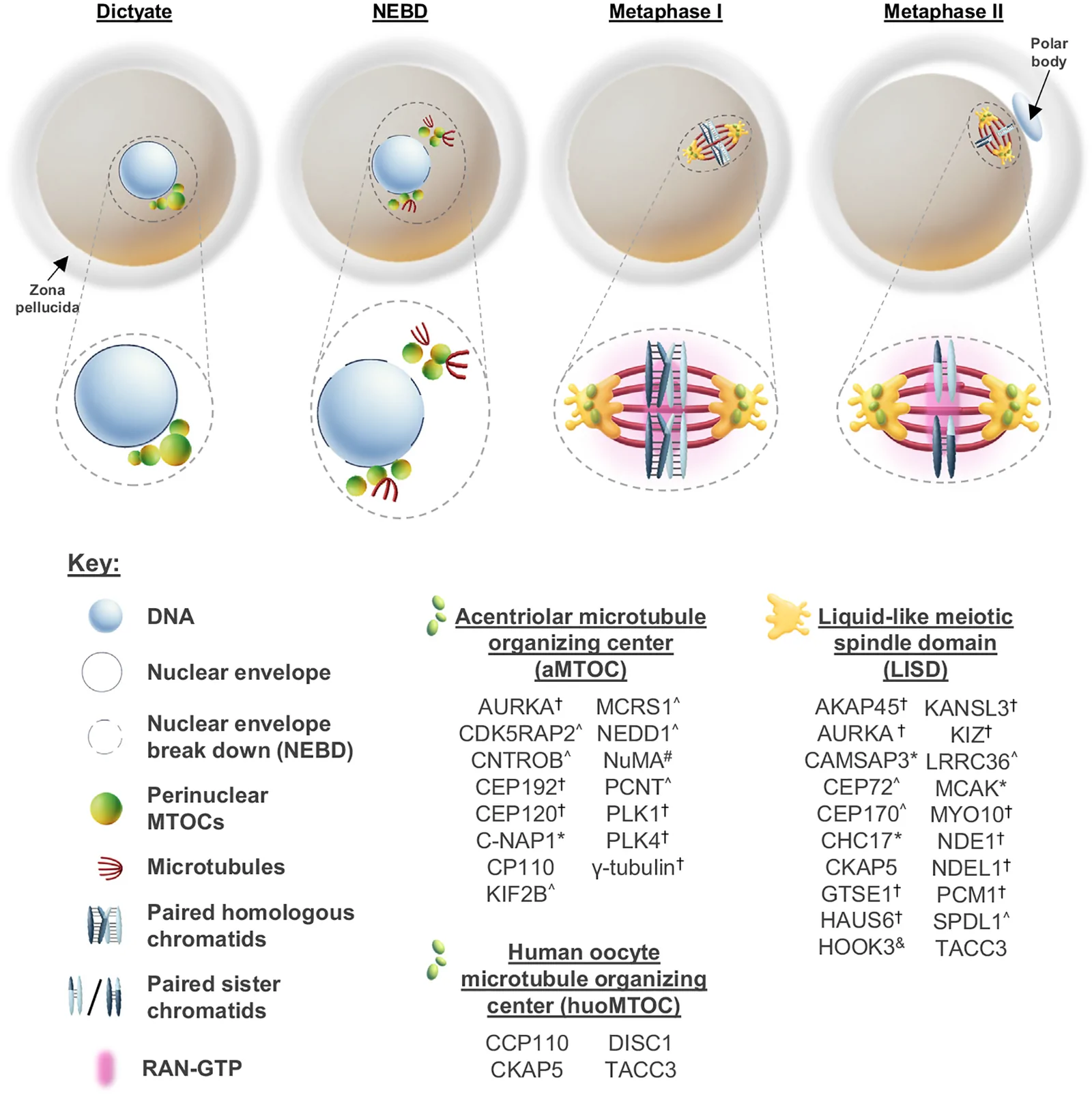
Formation of aMTOCs during oogenesis. During oogenesis, chromosome segregation is coordinated by aMTOCs. At dictyate, MTOC proteins assemble at the nuclear periphery. Upon NEBD, the cleavage of the linking protein C-NAP1 leads to the dissociation of MTOC proteins into multiple astral MTOCs. These astral MTOCs reform into two coordinated aMTOCs and LISDs by metaphase I in order to coordinate bipolar spindle formation and chromosome segregation. The aMTOC is also used to coordinate spindle pole formation at metaphase II. In humans, chromosome segregation in oocytes is facilitated by huoMTOCs. Components of the aMTOC and LISD observed in mouse have been characterized in human oocytes, and their observed localization is indicated by the following: ^†^ Present at human oocyte spindle microtubules; ^#^Present at human oocyte spindle poles; ^&^Present at human oocyte spindle periphery; ^Not present at any human oocyte spindle-related structure; *Not assessed in human oocytes. aMTOC: acentriolar MTOC; huoMTOC: human oocyte MTOC; LISD: liquid-like spindle domain; MTOC: microtubule-organizing center; NEBD: nuclear envelope break down.

Having eliminated its centrosomes, the oocyte must utilize an alternative way to organize its meiotic spindle. Mouse oocytes do so by creating dozens of small, functional aMTOCs scattered throughout their cytoplasms [112,122–124]. These aMTOCs, while lacking a centriolar core, are rich in many of the same PCM proteins found at canonical centrosomes, including the essential MT nucleator γ-tubulin and its associated γ-TuRC proteins, as well as scaffolding proteins like PCNT, CEP192, and NEDD1 (Figure 4) [24,124,125].

The assembly of the bipolar spindle from these diffuse aMTOCs is a multistep process of remarkable self-organization. A single cluster of PCM components resides closely juxtaposed to the nuclear envelope, which is also known as the germinal vesicle (GV). Following **germinal vesicle breakdown (GVBD)**, which marks the resumption of meiosis, MTs are first nucleated in the vicinity of the condensed chromosomes, forming a disorganized structure (Figure 4). The aMTOCs undergo stretching and subsequent fragmentation during GVBD, which requires the timely dissociation of the aMTOCs-linking proteins, C-NAP1 (also known as CEP250) and CEP152, from the PCM cluster via phosphorylation by PLK1 and CDK1, respectively [126–128]. PCM proteins such as PCNT and CEP192 serve as core scaffolds; their experimental depletion leads to the ablation of aMTOCs, delayed spindle formation, and severe chromosome alignment defects, confirming their essential structural role [128,129]. Within this immature aMTOC structure, a complex molecular dance, driven by motor proteins, begins to sort the components. Minus end-directed motors such as dynein and plus end-directed motors such as KIF11, KIF16A, KIF18A, and KIF20A work antagonistically to sort the randomly oriented MTs and transport the aMTOCs, which are attached to the MT minus ends, toward opposite ends of the structure [122,130–133]. NuMA, likely in conjunction with dynein, is implicated in anchoring MT minus ends to the aMTOCs accumulating at the poles [134]. Additionally, cytoplasmic MTOCs have been observed to help coordinate aMTOC formation and bipolar spindle orientation within mouse oocytes [135]. Over a period of several hours, this sorting process culminates in the clustering of aMTOCs into two focused poles, establishing a stable, bipolar spindle.

A crucial biomolecular condensate that functions as a stabilizer of mouse aMTOCs in oocytes is the **liquid-like spindle domain (LISD**; Figure 4). Formed through liquid–liquid phase separation, this membrane-less organelle is primarily assembled from a network of key proteins, including the scaffold protein TACC3, the AURKA, and the spindle-stabilizing protein, clathrin heavy chain 17 (CHC17) [24,136]. The process is initiated when AURKA phosphorylates TACC3, triggering its phase separation, which effectively creates a dynamic, liquid-like hub that concentrates essential factors for MT nucleation and organization [24]. By acting as a reservoir for these proteins, the LISD ensures the robust assembly of the bipolar spindle, which is required for accurate chromosome segregation during meiosis in the absence of traditional centrosomes [24,136]. In contrast to oocytes, spermatocytes do not appear to form LISD condensates [137]. However, AURKA and TACC3 associate with centrosomes and spindles during meiosis in spermatocytes and are important for bipolar spindle formation and stabilization [17,19,137]. Similar to that in mouse oocytes, meiotic spindle assembly in human oocytes is acentriolar [138]. The primary MTOC in human oocytes, known as the **human oocyte MTOC (huoMTOC)**, has been characterized during the GV and GVBD stages and has been shown to harbor many of the same proteins observed in mouse aMTOCs [139]. The huoMTOC appears to fragment and relocate to kinetochores after nuclear envelope breakdown, initiating MT nucleation. Disruption of huoMTOC components following GVBD severely impairs spindle assembly [124]. By metaphase I, the only proteins identified to localize to the huoMTOC are centriolar coiled-coil protein 110 (CCP110), cytoskeletal-associated protein 5 (CKAP5), disrupted in schizophrenia 1 (DISC1), and TACC3 [124]. However, additional investigation is still required to fully characterize huoMTOC-specific proteins and their roles at the different stages of meiotic division. Furthermore, the formation of bipolar spindles in human and other nonrodent mammalian oocytes relies heavily on NuMA [122]. NuMA becomes highly enriched at MT minus ends at the poles, recruiting the motor protein dynein to cluster these ends and organize the poles in the absence of aMTOCs. Depletion of NuMA or inhibition of dynein in human oocytes leads to defocused spindle poles, which is a distinct phenotype from the hyperfocused poles observed when NuMA is absent in mouse oocytes that harbor aMTOCs [122,134]. Another integral difference between mouse and human oocytes discovered is the expression of the minus end-directed kinesin, KIFC1 (also known as HSET). KIFC1 is not detectable in human oocytes, and this deficiency has been shown to contribute to the increased spindle instability observed in human oocytes compared with that in mouse oocytes [122,124].

These fundamental differences in organizing centers and stabilizing factors are reflected in the dramatically different timelines for spindle assembly. Mouse oocytes typically achieve bipolarity after 3–5 h, whereas human oocytes require ~16 h for the same process, further highlighting the unique challenges faced during human female meiosis.

### A two-pronged approach: aMTOC/huoMTOC- and chromosome-driven nucleation

While the aMTOC- and huoMTOC-driven mechanisms provide crucial organizing centers, spindle assembly in the oocyte is further bolstered by a distinct chromosome-driven pathway. This second pathway leverages the condensed chromosomes themselves as platforms for MT nucleation. This core mechanism is mediated by the Ran-GTPase system. The protein RCC1, which is bound to the chromatin, acts as a guanine nucleotide exchange factor for the small GTPase, Ran, creating a high concentration gradient of Ran bound to GTP (Ran-GTP) around the chromosomes [138,140–143]. This Ran-GTP gradient triggers the release of spindle assembly factors from inhibitory complexes in the cytoplasm [144]. A key spindle assembly factor released is TPX2, which, upon release, binds to and activates the kinase AURKA [101,145,146]. Activated AURKA can then promote local MT nucleation and stabilization, effectively turning the chromatin region into an additional MT-generating platform [24,147].

Therefore, oocyte spindle assembly is not solely reliant on one mechanism but emerges from the synergistic action of both pathways. However, the relative reliance on these two pathways differs significantly between species, potentially explaining variations in meiotic fidelity [112, 138,143]. Mouse oocytes utilize Ran-GTP synergistically with their dominant aMTOC-driven pathway, primarily for the massive MT increase after GVBD and for aMTOC coalescence [112,143]. Consequently, inhibiting Ran in mice impairs but does not abolish spindle formation if aMTOCs are functional [143]. By contrast, human oocytes, lacking prominent aMTOCs, rely more heavily on the chromosome-mediated Ran-GTP pathway, which drives MT nucleation via the huoMTOC structure at kinetochores [122,124,138]. This pathway drives MT nucleation directly at kinetochores, the first step in a unique, minor pole-mediated bipolarization mechanism. It has been established that the minus ends of these kinetochore-nucleated MTs coalesce to form transient ‘minor poles’ within kinetochore clusters [148]. These minor poles then slowly aggregate over a 7- to 9-h multipolar intermediate stage to form the final bipolar spindle. This assembly and stabilization are critically dependent on HAUS6 (for MT amplification), KIF11 (for elongation), and KIF18A (for stabilization) [148]. Mutations in the genes of the aforementioned proteins are linked to spindle bipolarization failure, aneuploidy, and female infertility [131,148]. This greater dependency on a kinetochore-driven, minor pole-mediated assembly process means that inhibiting the Ran pathway severely impairs or prevents spindle assembly in human oocytes [138]. This difference in reliance on the Ran-GTP chromosome-mediated pathway, and the subsequent, time-consuming aggregation of minor poles, contributes to the slower, less stable spindle assembly process and prolonged multipolar intermediate stage observed in humans compared with those in mice [122,124,138,148].

This species-specific balance between assembly mechanisms likely underlies the differing susceptibility to meiotic errors. A spindle built primarily ‘from the poles in’, as seen in mouse oocytes, may offer greater geometric stability from the outset. Conversely, a spindle built largely ‘from the chromosomes out’, as seen in human oocytes, is more structurally fluid and appears inherently unstable, increasing the risk of incorrect kinetochore–MT attachments that lead directly to aneuploidy. Understanding this mechanistic divergence is crucial for explaining the high rates of aneuploidy in human females and for interpreting mouse model data in the context of human reproduction.

## Concluding remarks and future perspectives

The fundamental biological processes governing MT organization during gametogenesis have profound clinical relevance. Failures in these intricate molecular machines are not abstract cellular defects; they are direct causes of human infertility, a condition with significant medical, social, and psychological impact. A detailed understanding of how these pathways can fail is essential for diagnosing the causes of infertility and for developing future therapeutic strategies.

Despite significant progress in understanding the MTOC strategies in gametogenesis, many fundamental questions remain, representing exciting frontiers for future research (see Outstanding questions). A deeper molecular understanding of these processes not only is crucial for advancing fundamental cell biology but also holds the promise of identifying novel diagnostic markers and therapeutic targets for human infertility.

Despite significant advances in the understanding of meiosis, several fundamental questions remain regarding the regulation of MTOCs and spindle assembly during gametogenesis. In male meiosis, a key puzzle lies in the regulation of the centrosome cycle. It remains unclear what precise molecular signals uncouple centriole duplication from the S-phase in spermatocytes. A deeper understanding is needed on how the activities of kinases such as AURKA, PLK1, and PLK4 are coordinated to execute two rounds of centriole duplication and maturation in the absence of a canonical cell cycle framework. While PLK4 is required for centriole duplication and maturation during mammalian spermatogenesis, PLK1 is necessary to block the second round of centriole duplication until anaphase I. Furthermore, PLK1 and AURKA are required for successful centriole maturation and separation prior to metaphase I. Compounding this complexity is the role of the ncMTOC pathway during meiotic divisions. The full functional significance of this pathway is yet to be determined, leaving open the question of whether it serves as a primary MTOC or possesses a specialized function in generating specific MT arrays, and how its activity is regulated and integrated with the canonical centrosome pathway. As centrosome components are not required for mediating chromosome segregation during spermatogenesis, it could be argued that the centrioles and PCM components are passengers that need to be inherited by each round spermatid to mediate their morphological transformations during spermiogenesis.

Shifting focus to female meiosis, the oocyte presents a different set of challenges, primarily centered on its aMTOCs. While key components such as PCNT and CEP192 have been identified, a complete inventory of the proteins constituting the oocyte aMTOC, examination of their dynamics, and determination of how they interact with one another are still needed. A central mystery is how these components assemble into a functional, MT-nucleating structure without a centriolar template to guide them. Furthermore, a critical area of investigation is the regulation of dual spindle-assembly pathways in oocytes. It is essential to understand how the balance is maintained between the aMTOC-driven and chromosome-driven MT nucleation pathways. Elucidating why human oocytes appear to rely more heavily on the chromosome-driven pathway is particularly urgent, as this dependency may be a key contributor to their characteristically high rate of meiotic errors.

Finally, from a clinical perspective, there is a pressing need for improved, noninvasive methods to assess gamete quality. Technologies such as polarized light or optical coherence microscopy, which can visualize the meiotic spindle in living oocytes, offer the potential to assess spindle morphology as a biomarker for developmental competence [149,150]. Developing and validating such tools could significantly improve embryo selection in assisted reproductive technologies and provide patients with more accurate prognostic information. Continued investigation into the dynamic scaffolds of the meiotic spindle is therefore essential, promising not only to unravel one of the cell biology’s most fascinating dichotomies but also to bring new hope to those affected by infertility.

## Supplementary Material

Supplemental Tables S1 and S2

Supplementary information associated with this article can be found online at https://doi.org/10.1016/j.treopn.2025.12.002.

## Significance

Successful reproduction requires healthy sperm and eggs. This review explains the dramatically different toolkits used by male and female germ cells to build the spindle, the molecular machine that separates chromosomes. Males use a canonical structure known as the centrosome, but recent work shows that they possess an alternative system. By contrast, females build a spindle from scratch. Understanding these strategies and how they fail is critical for diagnosing and treating infertility, a condition affecting millions globally.

## Box 1. The centrosome: an architectural review

Centrioles are the core structural unit of the centrosome. These cylindrical organelles comprise nine MT triplets arranged with ninefold symmetry around central structures (see Figure 1 in the main text and Table S1) [29]. The triplets consist of A, B, and C tubules, linked by A–C linkers [30–32]. The A tubule connects internally via pinhead structures (SAS4 and CEP44) to the proximal cartwheel, composed of SAS6, and other inner scaffold proteins, including centrins (CETN1–4), POC1/5, and FAM161A [30,31]. The distal end of a centriole is capped with cap proteins such as CP110 and CEP290, which limit its length unless removed during processes that require MT extension, such as ciliogenesis [30,31,33]. Asymmetric proteins such as centrobin and CEP120 transiently associate with daughter centrioles to regulate maturation [34–36].

Maturation and subsequent stabilization of the centriole require the acquisition of distal and subdistal appendages. Components of the distal appendage, including CEP164 and CEP83, form blade-like structures crucial for docking vesicles and initiating cilia formation [33,37]. Subdistal appendage proteins—including CEP170, ODF2, and ninein—anchor MTs, contribute to centrosome cohesion, and recruit PCM components [37,38].

The PCM is a highly ordered protein matrix that surrounds the centrioles, stabilizes centriole structures, and coordinates MT nucleation. Proteins typically fall into three general zones, with increasing distance from the centrioles (see Figure 1 in the main text and Table S2). CEP192 is proximal, followed by CEP152 and CDK5RAP2, with NEDD1 and γ-TuRCs located most distally [39–46]. The localization of CEP192 supports its role in recruiting the PCM and organizing MT nucleation [40–42]. Various PCM proteins, including NEDD1, CDK5RAP2, and pericentrin (PCNT), recruit and anchor γ-TuRCs [43,46–49]. PCNT forms a scaffold extending throughout the PCM [49]. Coiled-coil domains in many PCM proteins may promote phase separation-like interactions [39,50]. PCM accumulation is initially asymmetric on the mother centriole [51,52].

Centriole pairs are linked by fibers containing proteins including C-NAP1, rootletin, and CEP68 (see Figure 1 in the main text and Table S1) [53–55]. Cell cycle kinases, including PLK1 and AURKA, regulate linker disassembly during G2/M by phosphorylation, allowing KIF11 motors to push centrosomes apart [56,57]. Premature loss of centriole linkage and centrosome cohesion leads to defects in cell polarity, aberrant cell migration during development, and male infertility [17,58,59].

## Box 2. Spermiogenesis: sculpting the spermatozoon

Spermiogenesis is the postmeiotic transformation of round spermatids into specialized spermatozoa (Figure 2B). It involves four main phases, classified into 16 distinct steps in mice [67,68] and eight steps in humans [69] as follows:

1. Golgi phase (mouse steps 1–3; human step Sa): Acrosomal proteins required for fertilization accumulate from Golgi vesicles at the future anterior pole [67–69].
2. Cap phase (mouse steps 4–7; human steps Sb1 and Sb2): Transcription and translation are downregulated as chromatin begins to condense [70,71]. The acrosome grows to cover ~50% of the spermatid proximal end [68,72], and the centrosome attaches to the nucleus, migrating posteriorly to be distal of the acrosome [68,73,74].
3. Elongation (acrosome/tail) phase (mouse steps 8–12; human steps Sc and Sd1): Nuclear morphology changes as histones are replaced by transition proteins (TNP1/2) and then protamines (PRM1/2) for chromatin compaction [75,76]. Transient MT structures, known as the manchette and perinuclear ring, assemble [68,77,78]. The manchette, nucleated from the centriole adjunct, which is derived from the immature centriole, facilitates protein trafficking and nuclear shaping [68,77–80]. The perinuclear ring anchors the manchette [77,78]. Flagellum formation initiates from the basal body, which is derived from the mature centriole [79,81,82].
4. Maturation phase (mouse steps 13–16; human steps Sd2–Se2): The manchette disassembles, DNA is fully compacted, excess cytoplasm undergoes exocytosis, and spermatids are released into the lumen of the seminiferous tubules, and they migrate to the epididymis to undergo further maturation [72,74,83].

The mature spermatozoon consists of the head, neck, and flagellum (tail) (Figure I). The head contains highly condensed DNA capped by the acrosome. The neck forms the head-tail coupling apparatus (HTCA), connecting the head to the flagellum and housing centriole remnants. The flagellum comprises the mid-piece (axoneme, outer dense fibers, and mitochondrial sheath), principal piece (axoneme, outer dense fibers, and fibrous sheath), and terminal piece (axoneme only) [79,84]. The axoneme is typically a 9+2 microtubule arrangement [79].

Inheritance of a functional centrosome is critical. The mature centriole becomes the basal body anchoring the flagellum [79,82], while the immature centriole becomes the centriole adjunct, essential for the HTCA [79,81]. PCM proteins are repurposed into HTCA structures [21,79,85]. Mutations in centrosomal genes often cause acephalic (headless) spermatozoa and infertility [85–89].

Sperm centriole fate diverges evolutionarily; humans retain a canonical proximal centriole, but the distal one becomes atypical, whereas mice lose the canonical structure in both, forming adjunct and basal body remnants [21,73,79,81,85].

## Outstanding questions

What molecular signals uncouple centriole duplication from S-phase entry in spermatocytes, and how are the activities of Aurora kinase A, polo-like kinase (PLK)1, and PLK4 coordinated to execute two duplication rounds without a canonical cell cycle framework?

Given that PLK4 remains present at the centrosome throughout spermatogenesis, what meiosis-specific inhibitory mechanisms prevent centriole overduplication?

What roles do protein phosphatases and their regulators play to counterbalance the driving forces that cell cycle kinases perform during spermatogenesis, particularly in controlling the precise timing of centriole and centrosome maturation and separation?

What is the full functional significance of the noncentrosomal microtubule-organizing center (MTOC) pathway in male meiosis? Does it serve as a simple backup, or does it have an essential, specialized role in generating specific microtubule arrays, and how is it integrated with the centrosome?

How is the human oocyte MTOC (huoMTOC) initially assembled near the cortex, and what directs its migration and subsequent fragmentation for recruitment to kinetochores after nuclear envelope breakdown?

Can noninvasive imaging techniques (e.g., polarized light and optical coherence microscopy) reliably quantify subtle defects in spindle bipolarization dynamics or huoMTOC behavior in live human oocytes, which can serve as predictive biomarkers for aneuploidy risk and developmental competence?

## Glossary

Acentriolar microtubule-organizing center (aMTOC): a functional MTOC that lacks a centriolar core. In mouse oocytes, these structures are rich in PCM proteins and self-organize to form the meiotic spindle poles.
Aurora kinase A (AURKA): a key enzyme (kinase) that regulates centrosome maturation and separation in spermatocytes. In oocytes, it is essential for initiating LISD formation and promoting chromosome-driven spindle assembly.
Calmodulin-regulated spectrin-associated protein (CAMSAP): a family of proteins (CAMSAP1–3), which stabilize the minus ends of microtubules, contributing to the organization of noncentrosomal MT arrays.
Germinal vesicle breakdown (GVBD): the event marking the resumption of meiosis in oocytes, where the nuclear envelope (germinal vesicle) disassembles, allowing spindle components to interact with chromosomes.
Human oocyte microtubule-organizing center (huoMTOC): a unique structure in human oocytes, distinct from mouse aMTOCs, which lacks certain PCM proteins. It fragments and relocates to kinetochores to initiate microtubule nucleation.
KIF11: a plus end-directed motor protein (kinesin) that helps push spindle poles apart, essential for establishing bipolarity in both centrosomal and acentriolar spindle assembly pathways.
Liquid-like spindle domain (LISD): a biomolecular condensate in oocytes, formed by phase separation of proteins such as TACC3 and AURKA. It acts as a dynamic hub to concentrate and stabilize spindle assembly factors.
Meiosis: a specialized type of cell division that reduces the number of chromosomes by half, creating four haploid cells (gametes) from one diploid cell. It involves two consecutive rounds of division, meiosis I and meiosis II.
Microtubule-organizing center (MTOC): a general term for any cellular structure that serves as the primary site for nucleating (originating) and organizing microtubules, which form the spindle.
Noncentrosomal microtubule-organizing center (ncMTOC): an MTOC pathway that operates independently of the canonical centrosome. In spermatocytes, this pathway can compensate for centriole loss to ensure chromosome segregation.
Nuclear mitotic apparatus protein (NuMA): a protein that helps focus and anchor the minus ends of microtubules at spindle poles, crucial for organizing both centrosomal and acentriolar spindles.
Oogenesis: the process of female gamete (oocyte or egg) formation, which occurs in the ovaries. It involves meiosis, which is arrested at late prophase and then resumed prior to ovulation to produce a mature haploid egg poised for fertilization.
Pericentriolar material (PCM): the highly ordered protein matrix that surrounds the centrioles in a canonical centrosome. It contains the γ-TuRC and other factors necessary for microtubule nucleation.
Polo-like kinase (PLK)1: a critical cell cycle kinase. In spermatocytes, it regulates centrosome maturation, separation, and prevents premature centriole re-duplication during meiosis I. In oocytes, it facilitates aMTOC fragmentation.
PLK4: the master regulator kinase that initiates and controls centriole duplication. Its unique, meiosis-specific regulation in spermatocytes is a key aspect of gametogenesis.
Spermatogenesis: the process of male gamete (sperm) formation, which occurs in the testes. It involves meiosis and the complex postmeiotic remodeling process of spermiogenesis.
Spermiogenesis: the complex postmeiotic transformation process where a round spermatid undergoes dramatic morphological changes to become a functional spermatozoon with a head and flagellum.
Targeting protein for Xklp2 (TPX2): a key spindle assembly factor that, when released by Ran-GTP, activates AURKA and promotes microtubule nucleation and stabilization, especially in the chromosome-driven pathway.
γ-Tubulin ring complex (γ-TuRC): the primary protein complex responsible for nucleating microtubules. It is a key component of the PCM at canonical centrosomes and is also found in oocyte aMTOCs.
