# Supplementary material for: Divergent microtubule-organizing center strategies in mammalian gametogenesis
Source: Trends Open. Author manuscript; Available in PMC 2026 Sep 19. (PMC13588283; doi:10.1016/j.treopn.2025.12.002)
Supplement: Supplementary file 1 [file NIHMS2153398-supplement-Supplemental_Tables_S1_and_S2.docx]

**Table S1. Key centriole proteins, species orthologs, and localization.**

| **Organism** | | | |  |  |
| --- | --- | --- | --- | --- | --- |
| ***H. Sapiens*** | ***M. musculus*** | ***D. melanogaster*** | ***C. elegans*** | **Localization** | **Ref.** |
| α-tubulin | α-tubulin | α-tubulin | TBA-1 | MTs | [1] |
| β-tubulin | β-tubulin | β-tubulin | TBB-2 | MTs | [1] |
| CEP44 | CEP44 | - | - | Cartwheel | [2] |
| CEP120 | CEP120 | - | - | Cartwheel | [3] |
| CENPJ  (CPAP) | CENPJ (SAS4) | Sas-4 | SAS-4 | Cartwheel | [4] |
| SAS6 | SAS6 | Sas6 | SAS-6 | Cartwheel | [5] |
| STIL | STIL | Ana2 | SAS-5 | Cartwheel | [6] |
| CETN1 | CETN1 | CG17493 | - | Centriole inner wall | [7] |
| CETN2 | CETN2 |  | - | Centriole inner wall | [8] |
| CETN3 | CETN3 | - | - | Centriole inner wall | [9] |
| CETN4P | CETN4 | CG31802 | - | Centriole inner wall | [10] |
| FAM161A | FAM161A | Fam161 | F58A4.1 | Centriole inner wall | [11] |
| POC1 | POC1 | Poc1 | - | Centriole inner wall | [12] |
| POC5 | POC5 | - | - | Centriole inner wall | [13] |
| SPICE | SPICE | - | - | Centriole inner wall | [14] |
| CEP97 | CEP97 | Cep97 | CEP97 | Distal cap | [15] |
| CEP162 | CEP162 | CG42699 | - | Distal cap | [16] |
| CEP290 | CEP290 | - | - | Distal cap | [17] |
| CP110 | CP110 | Cp110 | - | Distal cap | [15] |
| ANKRD26 | ANKRD26 | CG8679 | - | Distal appendage | [18] |
| CEP83 | CEP83 | CG5348 | F57B9.1 | Distal appendage | [19] |
| CEP89 | CEP89 | Cep89 | - | Distal appendage | [20] |
| CEP164 | CEP164 | Cep164 | - | Distal appendage | [21] |
| FBF1 | FBF1 | Twy | DYF-19 | Distal appendage | [19] |
| OFD1 | OFD1 | - | - | Distal appendage | [22] |
| SCLT1 | SCLT1 | - | - | Distal appendage | [19] |
| TTBK2 | TTBK2 | Asator | TTBK-7 | Distal appendage | [23] |
| CCDC120 | CCDC120 | Ccdc120 | - | Subdistal appendage | [24] |
| CNTRL (CEP110) | CNTRL (CEP110) | - | - | Subdistal appendage | [25] |
| CEP43 (FOP) | CEP43 (FOP) | - | - | Subdistal appendage | [26] |
| CEP128 | CEP128 | CG14771 | - | Subdistal appendage | [27] |
| CEP170 | CEP170 | - | - | Subdistal appendage | [28] |
| CEP350 | CEP350 | - | - | Subdistal appendage | [29] |
| NINEIN | NINEIN | Ninein | NOCA-2 | Subdistal appendage | [25] |
| ODF2 | ODF2 | Odf2 | - | Subdistal appendage | [30] |
| CCDC102B | CCDC102B | CG13764 | NSY-1 | Linking fiber | [31] |
| CNTLN | CNTLN | - | - | Linking fiber | [32] |
| CEP68 | CEP68 | CG14934 | - | Linking fiber | [33] |
| C-NAP1 (CEP250) | C-NAP1 | - | - | Linking fiber | [34] |
| LRRC45 | LRRC45 | - | - | Linking fiber | [35] |
| ROOTLETIN | ROOTLETIN | Root | IFI-1 | Linking fiber | [36] |
| β-catenin | β-catenin | Arm | HMP-2 | Linking fiber | [37] |

**Table S2. Key centrosome-associated protein species, orthologs, and centrosomal localization.**

| **Organism** | | | |  |  |
| --- | --- | --- | --- | --- | --- |
| ***H. Sapiens*** | ***M. musculus*** | ***D. melanogaster*** | ***C. elegans*** | **Localization** | **Ref.** |
| CNTROB | CNTROB | Cnb | - | Torus/PCM | [38] |
| CEP135 | CEP135 | Bld10 | - | Torus/PCM | [39] |
| PLK4 | PLK4 | Sak | ZYG-1 | Torus/PCM | [40] |
| AKAP450 | PCNT | Dplp | - | PCM | [41] |
| AURKA | AURKA | Aurka | AIR-2 | PCM | [42] |
| CKAP5 (chTOG) | CKAP5 (chTOG) | Msps | ZYG-9 |  | [43] |
| CEP152 | CEP152 | Asl | - | PCM | [44] |
| CEP192 | CEP192 | Dspd2 | SPD-2 | PCM | [45] |
| CEP215 | CDK5RAP2 (CEP215) | Cnn | SPD-5 | PCM | [46] |
| CEP295 | CEP295 | Ana1 | SAS-7 | PCM | [47] |
| GCP2 | GCP2 | Grip84 | - | PCM | [48] |
| GCP3 | GCP3 | Grip91 | - | PCM | [48] |
| GCP4 | GCP4 | Grip75 | GTAP-1 and GTAP-2 | PCM | [48] |
| GCP5 | GCP5 | Grip128 |  | PCM | [48] |
| GCP6 | GCP6 | Grip163 |  | PCM | [48] |
| MZT2A  (GCP8) | MZT2  (GCP8) | Msps | ZYG-9 | PCM | [49] |
| NEDD1 (GCP7) | NEDD1 | Dgrip71 | - | PCM | [50] |
| PCM1 | PCM1 | Cmb | PCM-1 | PCM | [51] |
| PLK1 | PLK1 | Polo | PLK-1 | PCM | [52] |
| PPP2ca | PPP2ca | PP2A | LET-92 | PCM | [53] |
| PPP2r1a | PPP2r1a | PP2A-B | SUR-6 | PCM | [53] |
| PP4c | PP4c | PP4 | PPh4.1 | PCM | [54] |
| TACC2 | TACC3 | D-Tacc | TAC-1 | PCM | [55] |
| TACC3 | TACC3 | Tacc |  | PCM | [55] |
| γ-tubulin | γ-tubulin | γ-tubulin | TBG-1 | PCM | [56] |
| CAMSAP1 | CAMSAP1 | Patronin | PTRN-1 | **ncMTOC** (PCM) | [57] |
| CAMSAP2 | CAMSAP2 |  |  | **ncMTOC** (PCM) | [57] |
| CAMSAP3 | CAMSAP3 |  |  | **ncMTOC** (PCM) | [57] |
| KIF11  (EG5) | KIF11 | KLP61F | BMK-1 | **ncMTOC** (PCM) | [58] |
| NuMA | NuMA | Mud | NuMA | **ncMTOC** (PCM) | [59] |
| TPX2 | TPX2 | D-Tpx2 | TPX2 | **ncMTOC** (PCM) | [60] |

**References for Supplemental Table S1 and S2**

1	Wade, R.H. and Chrétien, D. (1993) Cryoelectron microscopy of microtubules. *J. Struct. Biol.* 110, 1–27

2	Zhang, D. *et al.* (2024) CEP44 is required for maintaining centriole duplication and spindle integrity. DOI: 10.7554/eLife.94405.1

3	Betleja, E. *et al.* (2018) A novel Cep120-dependent mechanism inhibits centriole maturation in quiescent cells. *Elife* 7,

4	Kohlmaier, G. *et al.* (2009) Overly long centrioles and defective cell division upon excess of the SAS-4-related protein CPAP. *Curr. Biol.* 19, 1012–1018

5	Kitagawa, D. *et al.* (2011) Structural basis of the 9-fold symmetry of centrioles. *Cell* 144, 364–375

6	Vulprecht, J. *et al.* (2012) STIL is required for centriole duplication in human cells. *J. Cell Sci.* 125, 1353–1362

7	Avasthi, P. *et al.* (2013) Germline deletion of Cetn1 causes infertility in male mice. *J. Cell Sci.* 126, 3204–3213

8	Salisbury, J.L. *et al.* (2002) Centrin-2 is required for centriole duplication in mammalian cells. *Curr. Biol.* 12, 1287–1292

9	Middendorp, S. *et al.* (2000) A role for centrin 3 in centrosome reproduction. *J. Cell Biol.* 148, 405–416

10	Gavet, O. *et al.* (2003) Centrin4p, a novel mammalian centrin specifically expressed in ciliated cells. *Mol. Biol. Cell* 14, 1818–1834

11	Zach, F. and Stöhr, H. (2014) FAM161A, a novel centrosomal-ciliary protein implicated in autosomal recessive retinitis pigmentosa. *Adv. Exp. Med. Biol.* 801, 185–190

12	Sala, C. *et al.* (2024) An interaction network of inner centriole proteins organised by POC1A-POC1B heterodimer crosslinks ensures centriolar integrity. *Nat. Commun.* 15, 9857

13	Azimzadeh, J. *et al.* (2009) hPOC5 is a centrin-binding protein required for assembly of full-length centrioles. *J. Cell Biol.* 185, 101–114

14	Archinti, M. *et al.* (2010) SPICE--a previously uncharacterized protein required for centriole duplication and mitotic chromosome congression. *J. Cell Sci.* 123, 3039–3046

15	Aydogan, M.G. *et al.* (2022) Centriole distal-end proteins CP110 and Cep97 influence centriole cartwheel growth at the proximal end. *J. Cell Sci.* 135,

16	Wang, W.-J. *et al.* (2013) CEP162 is an axoneme-recognition protein promoting ciliary transition zone assembly at the cilia base. *Nat. Cell Biol.* 15, 591–601

17	Kim, J. *et al.* (2008) CEP290 interacts with the centriolar satellite component PCM-1 and is required for Rab8 localization to the primary cilium. *Hum. Mol. Genet.* 17, 3796–3805

18	Evans, L.T. *et al.* (2021) ANKRD26 recruits PIDD1 to centriolar distal appendages to activate the PIDDosome following centrosome amplification. *EMBO J.* 40, e105106

19	Tanos, B.E. *et al.* (2013) Centriole distal appendages promote membrane docking, leading to cilia initiation. *Genes Dev.* 27, 163–168

20	Chong, W.M. *et al.* (2020) Super-resolution microscopy reveals coupling between mammalian centriole subdistal appendages and distal appendages. *Elife* 9,

21	Graser, S. *et al.* (2007) Cep164, a novel centriole appendage protein required for primary cilium formation. *J. Cell Biol.* 179, 321–330

22	Singla, V. *et al.* (2010) Ofd1, a human disease gene, regulates the length and distal structure of centrioles. *Dev. Cell* 18, 410–424

23	Lo, C.-H. *et al.* (2019) Phosphorylation of CEP83 by TTBK2 is necessary for cilia initiation. *J. Cell Biol.* 218, 3489–3505

24	Huang, N. *et al.* (2017) Hierarchical assembly of centriole subdistal appendages via centrosome binding proteins CCDC120 and CCDC68. *Nat. Commun.* 8, 15057

25	Ou, Y.Y. *et al.* (2002) CEP110 and ninein are located in a specific domain of the centrosome associated with centrosome maturation. *J. Cell Sci.* 115, 1825–1835

26	Lee, J.Y. and Stearns, T. (2013) FOP is a centriolar satellite protein involved in ciliogenesis. *PLoS One* 8, e58589

27	Kashihara, H. *et al.* (2019) Cep128 associates with Odf2 to form the subdistal appendage of the centriole. *Genes Cells* 24, 231–243

28	Guarguaglini, G. *et al.* (2005) The forkhead-associated domain protein Cep170 interacts with Polo-like kinase 1 and serves as a marker for mature centrioles. *Mol. Biol. Cell* 16, 1095–1107

29	Mojarad, B.A. *et al.* (2017) CEP19 cooperates with FOP and CEP350 to drive early steps in the ciliogenesis programme. *Open Biol* 7,

30	Ishikawa, H. *et al.* (2005) Odf2-deficient mother centrioles lack distal/subdistal appendages and the ability to generate primary cilia. *Nat. Cell Biol.* 7, 517–524

31	Xia, Y. *et al.* (2018) CCDC102B functions in centrosome linker assembly and centrosome cohesion. *J. Cell Sci.* 131,

32	Fang, G. *et al.* (2014) Centlein mediates an interaction between C-Nap1 and Cep68 to maintain centrosome cohesion. *J. Cell Sci.* 127, 1631–1639

33	Pagan, J.K. *et al.* (2015) Degradation of Cep68 and PCNT cleavage mediate Cep215 removal from the PCM to allow centriole separation, disengagement and licensing. *Nat. Cell Biol.* 17, 31–43

34	Mayor, T. *et al.* (2000) The centrosomal protein C-Nap1 is required for cell cycle-regulated centrosome cohesion. *J. Cell Biol.* 151, 837–846

35	He, R. *et al.* (2013) LRRC45 is a centrosome linker component required for centrosome cohesion. *Cell Rep.* 4, 1100–1107

36	Bahe, S. *et al.* (2005) Rootletin forms centriole-associated filaments and functions in centrosome cohesion. *J. Cell Biol.* 171, 27–33

37	Bahmanyar, S. *et al.* (2008) beta-Catenin is a Nek2 substrate involved in centrosome separation. *Genes Dev.* 22, 91–105

38	Ogungbenro, Y.A. *et al.* (2018) Centrobin controls primary ciliogenesis in vertebrates. *J. Cell Biol.* 217, 1205–1215

39	Hardy, T. *et al.* (2014) Multisite phosphorylation of C-Nap1 releases it from Cep135 to trigger centrosome disjunction. *J. Cell Sci.* 127, 2493–2506

40	Holland, A.J. *et al.* (2012) Polo-like kinase 4 controls centriole duplication but does not directly regulate cytokinesis. *Mol. Biol. Cell* 23, 1838–1845

41	Keryer, G. *et al.* (2003) Part of Ran is associated with AKAP450 at the centrosome: involvement in microtubule-organizing activity. *Mol. Biol. Cell* 14, 4260–4271

42	Chou, E.-J. *et al.* (2016) Phosphorylation of CPAP by Aurora-A Maintains Spindle Pole Integrity during Mitosis. *Cell Rep.* 14, 2975–2987

43	Gergely, F. *et al.* (2003) The ch-TOG/XMAP215 protein is essential for spindle pole organization in human somatic cells. *Genes Dev.* 17, 336–341

44	Cizmecioglu, O. *et al.* (2010) Cep152 acts as a scaffold for recruitment of Plk4 and CPAP to the centrosome. *J. Cell Biol.* 191, 731–739

45	Kuriyama, R. and Fisher, C.R. (2020) A novel mitosis-specific Cep215 domain interacts with Cep192 and phosphorylated Aurora A for organization of spindle poles. *J. Cell Sci.* 133,

46	Graser, S. *et al.* (2007) Cep68 and Cep215 (Cdk5rap2) are required for centrosome cohesion. *J. Cell Sci.* 120, 4321–4331

47	Izquierdo, D. *et al.* (2014) Stabilization of cartwheel-less centrioles for duplication requires CEP295-mediated centriole-to-centrosome conversion. *Cell Rep.* 8, 957–965

48	Zheng, Y. *et al.* (1995) Nucleation of microtubule assembly by a gamma-tubulin-containing ring complex. *Nature* 378, 578–583

49	Teixidó-Travesa, N. *et al.* (2010) The gammaTuRC revisited: a comparative analysis of interphase and mitotic human gammaTuRC redefines the set of core components and identifies the novel subunit GCP8. *Mol. Biol. Cell* 21, 3963–3972

50	Zhang, X. *et al.* (2009) Sequential phosphorylation of Nedd1 by Cdk1 and Plk1 is required for targeting of the gammaTuRC to the centrosome. *J. Cell Sci.* 122, 2240–2251

51	Dammermann, A. and Merdes, A. (2002) Assembly of centrosomal proteins and microtubule organization depends on PCM-1. *J. Cell Biol.* 159, 255–266

52	Lee, K. and Rhee, K. (2011) PLK1 phosphorylation of pericentrin initiates centrosome maturation at the onset of mitosis. *J. Cell Biol.* 195, 1093–1101

53	Meraldi, P. and Nigg, E.A. (2001) Centrosome cohesion is regulated by a balance of kinase and phosphatase activities. *J. Cell Sci.* 114, 3749–3757

54	Toyo-oka, K. *et al.* (2008) Protein phosphatase 4 catalytic subunit regulates Cdk1 activity and microtubule organization via NDEL1 dephosphorylation. *J. Cell Biol.* 180, 1133–1147

55	Peset, I. and Vernos, I. (2008) The TACC proteins: TACC-ling microtubule dynamics and centrosome function. *Trends Cell Biol.* 18, 379–388

56	Pereira, G. and Schiebel, E. (1997) Centrosome-microtubule nucleation. *J. Cell Sci.* 110 ( Pt 3), 295–300

57	Hendershott, M.C. and Vale, R.D. (2014) Regulation of microtubule minus-end dynamics by CAMSAPs and Patronin. *Proc. Natl. Acad. Sci. USA* 111, 5860–5865

58	Mardin, B.R. and Schiebel, E. (2012) Breaking the ties that bind: new advances in centrosome biology. *J. Cell Biol.* 197, 11–18

59	van Toorn, M. *et al.* (2023) NuMA deficiency causes micronuclei via checkpoint-insensitive k-fiber minus-end detachment from mitotic spindle poles. *Curr. Biol.* 33, 572–580.e2

60	Holder, J. *et al.* (2024) CEP192 localises mitotic Aurora-A activity by priming its interaction with TPX2. *EMBO J.* 43, 5381–5420
